# Enhanced Food Anticipatory Activity Associated with Enhanced Activation of Extrahypothalamic Neural Pathways in Serotonin_2C_ Receptor Null Mutant Mice

**DOI:** 10.1371/journal.pone.0011802

**Published:** 2010-07-27

**Authors:** Jennifer L. Hsu, Lisa Yu, Elinor Sullivan, Melodi Bowman, Ralph E. Mistlberger, Laurence H. Tecott

**Affiliations:** 1 Gladstone Institute of Neurological Disease, San Francisco, California, United States of America; 2 Department of Psychiatry, University of California San Francisco, San Francisco, California, United States of America; 3 Department of Neuroscience, Oregon National Primate Research Center, Beaverton, Oregon, United States of America; 4 Department of Psychology, Simon Fraser University, Burnaby, Canada; Pennsylvania State University, United States of America

## Abstract

The ability to entrain circadian rhythms to food availability is important for survival. Food-entrained circadian rhythms are characterized by increased locomotor activity in anticipation of food availability (food anticipatory activity). However, the molecular components and neural circuitry underlying the regulation of food anticipatory activity remain unclear. Here we show that serotonin_2C_ receptor (5-HT2CR) null mutant mice subjected to a daytime restricted feeding schedule exhibit enhanced food anticipatory activity compared to wild-type littermates, without phenotypic differences in the impact of restricted feeding on food consumption, body weight loss, or blood glucose levels. Moreover, we show that the enhanced food anticipatory activity in 5-HT2CR null mutant mice develops independent of external light cues and persists during two days of total food deprivation, indicating that food anticipatory activity in 5-HT2CR null mutant mice reflects the locomotor output of a food-entrainable oscillator. Whereas restricted feeding induces c-*fos* expression to a similar extent in hypothalamic nuclei of wild-type and null mutant animals, it produces enhanced expression in the nucleus accumbens and other extrahypothalamic regions of null mutant mice relative to wild-type subjects. These data suggest that 5-HT2CRs gate food anticipatory activity through mechanisms involving extrahypothalamic neural pathways.

## Introduction

Multiple aspects of physiology and behavior exhibit circadian rhythmicity [1]. These circadian rhythms are driven by an endogenous molecular clock located in the suprachiasmatic nucleus (SCN) and entrains to light/dark cues from the environment [2]. However, many species have also evolved mechanisms for the entrainment of circadian rhythms to other environmental cues important for survival, such as food availability. Restricted feeding schedules, in which food availability is fixed to a particular time of day, robustly induce preprandial increases in locomotor activity (food anticipatory activity; FAA), body temperature, and corticosterone release in anticipation of the daily scheduled meal [3], [4], [5].

There is strong evidence that FAA reflects the behavioral output of a food-entrainable circadian oscillator (FEO) [6], [7]. FAA entrains to feeding cycles of roughly 24 hours, but not to 19-hour or 29-hour cycles [3], [8]. In addition, FAA can develop in the absence of external lighting schedules [9], [10], [11]. Furthermore, once established, FAA persists after two or more days of total food deprivation [8], indicating that FAA is not driven by cycles of energy depletion and restoration imposed by the scheduled meal.

The neural and molecular mechanisms underlying FAA remain unclear [3], [5]. c-*fos* mapping and other studies have identified numerous sites in the hypothalamus, limbic system and elsewhere that may potentially regulate FAA [12], [13], [14], [15], [16]. Lesions at some of these sites, including the nucleus accumbens (NAc), dorsomedial hypothalamus (DMH) and lateral hypothalamic area (LHA), have been reported to attenuate the magnitude of FAA [14], [17], [18], [19]; however, other studies indicate that lesions to the above brain regions do not eliminate FAA in all measures of anticipatory behavior [20], [21], [22], [23], [24], [25].

Brain serotonin systems regulate many aspects of behavior including locomotor activity and food intake; however, there are a limited number of studies investigating serotonergic regulation of FAA. Impaired FAA in aged rats can be restored to levels comparable to young animals by treatment with non-selective 5-HT2R antagonists [26], suggesting the possibility that these receptors may regulate FAA. Of the three 5-HT2R subtypes (5-HT2AR, 5-HT2BR, 5-HT2CR), the 5-HT2CR warrants particular attention as a potential regulator of FAA. 5-HT2CRs are widely expressed in multiple brain structures, including the DMH, LHA, and NAc [27], [28]. Furthermore, 5-HT2CRs are strongly implicated in the regulation of feeding and locomotor activity [29], [30], [31]. In the present study, we investigate the impact of a null mutation of the 5-HT2CR on the regulation of FAA and its underlying neural circuitry.

## Materials and Methods

### Animals

5-HT2CR null mutant mice were generated as described previously [29]. The mutation is congenic on a C57BL/6 background. 12–16 week old male mutant mice and their wild-type littermates were used for this study. All experiments were performed according to guidelines of the National Institute of Health *Guide for Care and Use of Laboratory Animals*, and approved by the University of California San Francisco Committee on Animal Research.

### Restricted Feeding protocols

#### Experiment 1

All mice were housed with a 12 hour light: 12 hour dark (LD) cycle with light (100 lux) on at 7 AM (ZT0). Wild-type (WT) and 5-HT2CR null mutant (knockout; KO) mice were individually housed in standard polypropylene cages (45×24×17 cm) surrounded by 8×4 photobeam activity monitoring brackets (PAS Flexfield, San Diego Instruments). All animals were acclimated to these cages for 10–12 days prior to the restricted feeding schedule, with *ad libitum* food (Purina PicoLab 5058) and water access, and with food weighed every day at ZT4 and ZT8. Food restricted animals (RF group, *n* = 13 WT, *n* = 11 KO) were then food deprived for 20 hours and subsequently limited to 4 hours of restricted food access each day from ZT4-8 for 14 days, while control animals remained on an *ad libitum* feeding schedule (AL group, *n* = 13 WT, *n* = 11 KO). Main behavioral measurements were daily food intake and FAA (activity during the two hours prior to food availability). Body weight measurements were taken at the end of the baseline period and at the end of the study.

#### Experiment 2

To determine whether FAA occurred independently of oscillations in energy status imposed by the restricted feeding schedule, WT (*n* = 16) and KO (*n* = 16) animals were subjected to the restricted feeding protocol as above, but with two additional days of total food deprivation following the 14 days of restricted feeding. Behavioral measurements and body weight measurements were taken as above.

#### Experiment 3

To determine whether FAA occurred independent of light cues, WT (*n* = 15) and KO (*n* = 16) animals were subjected to the same restricted feeding protocol as in Experiment 1, except that the housing room was switched to dim red light at ZT12 the day prior to restricted feeding and maintained in dim red light for the duration of the restricted feeding phase of the experiment. Behavioral measurements and body weight measurements were obtained as described above.

### Tissue preparation

For c-*fos* gene expression studies, additional groups of mice were subjected to the same restricted feeding paradigm as in Experiment 1. Mice were sacrificed by decapitation at ZT4 (*n* = 8/group) or ZT13 (*n* = 5/group) on day 14 of restricted feeding. Brains were rapidly dissected and flash frozen in an isopentane/dry ice bath (−45 degrees Celsius) and stored at −80 degrees Celsius. Mice taken at ZT13 were sacrificed under dim red light conditions (Kodak Safelight with GBX-2 filter, 15W). Coronal brain sections (20 microns) were collected using a cryostat (Leica 1950) onto Superfrost Plus slides (Fisher) and stored at −80 degrees Celsius. Sections were collected in six series such that sections within each series were spaced 120 microns apart.

### In situ hybridization

A 0.5 kb fragment of mouse c-*fos* cDNA was used to generate the digoxigenin-labeled antisense riboprobe. Sections were fixed in ice-cold 4% paraformaldehyde for 30 minutes, rinsed with PBS, acetylated with .25% acetic anhydride in 0.1M triethanolamine for 10 minutes, rinsed with PBS, and then equilibrated with hybridization buffer for 30 minutes. Sections were then hybridized overnight with the c-*fos* antisense riboprobe (200 ng/ml) in a humidified chamber at 62 degrees Celsius. Following hybridization, sections were rinsed with 0.1X SSC at 72 degrees Celsius for 1 hour, and incubated with 1∶5000 alkaline phosphatase conjugated anti-digoxigenin antibody (Roche) overnight at 4 degrees Celsius. Slides were then incubated in BM Purple (Roche) overnight, rinsed with 10 mM Tris, 1 mM EDTA pH 8.0 for 10 minutes, dehydrated in an ascending ethanol series, and coverslipped with Aquamount.

### Quantitation of c-fos expression

Measurements were taken from the complete rostrocaudal extent of the arcuate nucleus (ARC), suprachiasmatic nucleus (SCN), and nucleus accumbens (NAc). Measurements for the dorsomedial nucleus of the hypothalamus (DMH), lateral hypothalamic area (LHA), ventral posterior thalamus (VPT) and barrel cortex were taken from sections corresponding to AP coordinates −1.58 to −1.94 from Bregma[32]. For all regions of interest (ROI) except the barrel cortex, adjacent series of sections stained with cresyl violet were used to determine outlines of the ROI according to the Mouse Brain atlas [32]. These outlines were then digitally transferred to photomicrographs from c-*fos* stained sections using visual landmarks as a guide for transfer. To delineate the barrel cortex, a standard 0.75 mm×0.75 mm square was centered on the cortex 3 mm from the midline, according to the Mouse Brain atlas [32]. Expression values from ROI were quantified by densitometry using ImageJ (http://rsb.info.nih.gov/ij/) and normalized to background as determined by calculating the average of three 50×50 micron sample regions from the same tissue section which contained no detectable staining. Expression =  (mean OD_ROI_-mean OD_Background_)/mean OD_Background_.

### Data analysis

Locomotor activity and food intake measurements were averaged over the baseline period and over the second week of restricted feeding (RW2). c-*fos* gene expression values for each data set were normalized to the average WT-AL value. Regression analysis was used to determine correlations between c-*fos* gene expression and locomotor activity in the hour leading up to the sacrifice time, also normalized to the average WT-AL group value. All data sets were tested for normality of distribution using the Kolmogorov-Smimov and Shapiro-Wilk tests. Data sets that failed both tests were analyzed using the non-parametric Mann-Whitney U test with the following comparisons: WT versus KO, Restricted versus Ad Lib, Restricted KO vs. Restricted WT. Otherwise, data sets were analyzed using ANOVA unless otherwise noted. For locomotor activity and food intake measurements, repeated measures 2×2×2 ANOVA were used to determine the effects of experimental period (RW2 vs. baseline), genotype (WT vs. KO), feeding condition (RF vs. AL), and their interactions on behavioral measures. For endpoint measures such as body weight, blood glucose levels, and c-*fos* gene expression, 2×2 ANOVA was used to determine the effects of genotype and feeding condition. Significance level was set at *p*<0.05.

## Results

### Restricted feeding has a similar impact on measures related to energy balance in WT and KO mice

**Figure 1:**
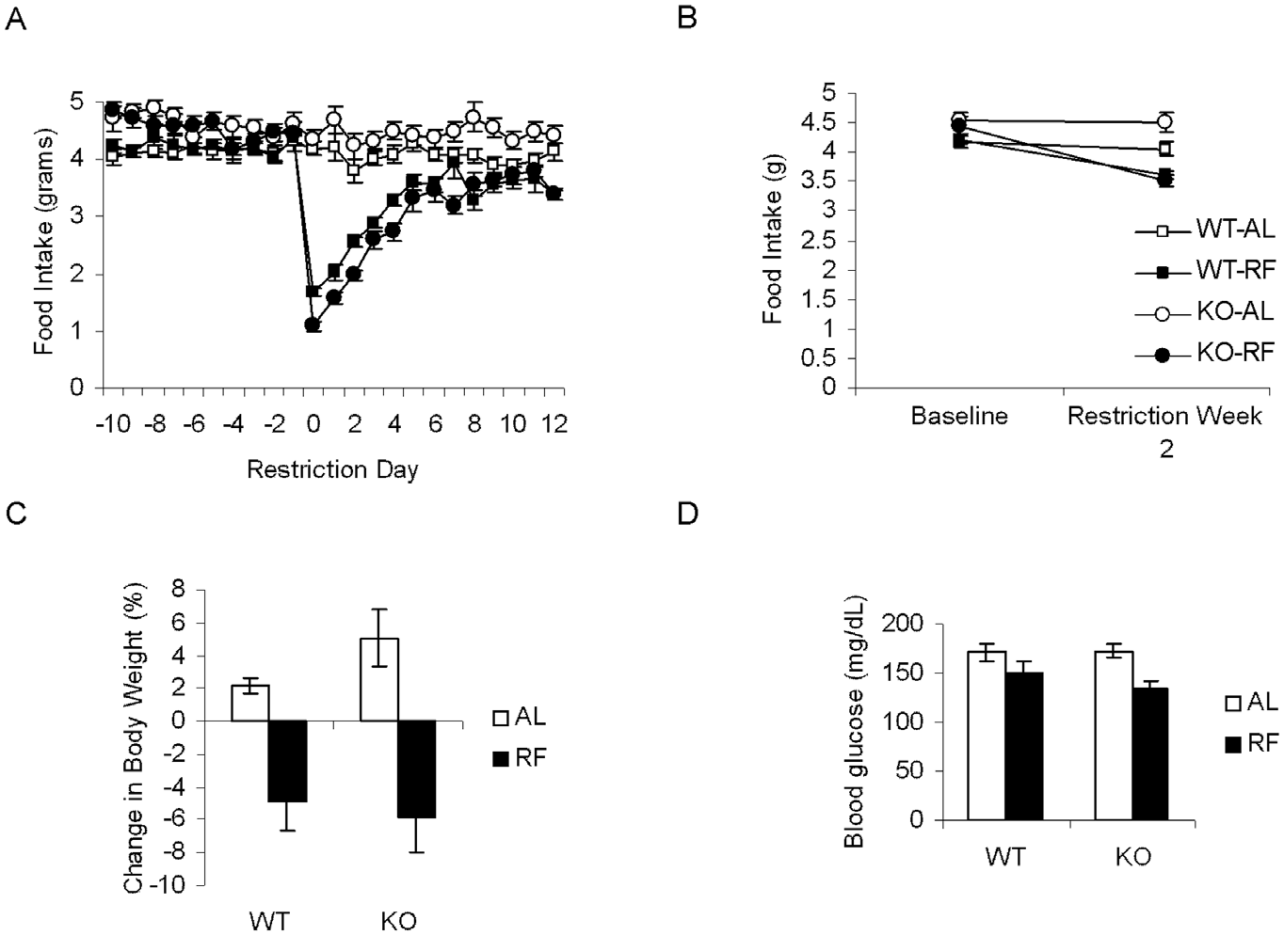
Restricted feeding induces similar changes in food intake, body weight, and blood glucose for both WT and KO mice. A. Daily food intake of WT (squares) or KO (circles) mice assigned to either *ad libitum* (white) or restricted feeding (black) groups. B. Daily food intake, averaged over the baseline period and second week of restricted feeding. C. Percent change in body weight, calculated from body weight measurements at the end of the baseline period and at the end of fourteen days of restricted feeding. White bars indicate *ad libitum* feeding conditions, black bars indicate restricted feeding conditions. D. Blood glucose levels measured at the end of fourteen days of restricted feeding. Values represent means ±S.E.M.

To determine whether the restricted feeding paradigm differentially affected measures related to energy balance in WT and KO mice, we assessed the impact of restricted feeding on daily food intake, body weight, and blood glucose levels. Baseline measures of daily food intake were not significantly different between WT and KO mice (2×2 ANOVA for average baseline food intake: effect of genotype, *p* = 0.568, effect of feeding condition, *p* = 0.912, genotype x feeding condition interaction, *p* = 0.758, Figure 1A). On the first day of restricted feeding, there was a significant decline in food intake for restricted mice of both genotypes, with restricted KO mice displaying greater decreases in food intake compared to WT mice (Figure 1A). However, both restricted WT and KO mice recovered their food intake at similar rates during the first week of restricted feeding (Figure 1A), and achieved steady levels of food intake by the second week of restricted feeding without phenotypic differences (2×2×2 RM ANOVA: experiment phase (RW2 vs. Baseline), *p* = 0.081, experiment phase x genotype, *p* = 0.569, experiment phase x feeding condition, *p* = 0.001, experiment phase x genotype x feeding condition, *p* = 0.871, Figures 1A–1B).

Body weight measurements taken at the end of the baseline experimental period and the end of the restricted feeding period were used to calculate the percent change in body weight. KO mice subjected to *ad libitum* feeding conditions gained slightly more weight than WT counterparts, but restricted KO mice lost a similar percentage of body weight as their WT counterparts (2×2 ANOVA: effect of genotype, *p* = 0.474, effect of feeding condition, *p*<0.001, genotype x feeding condition, *p* = 0.302, Figure 1C). We also measured blood glucose levels at the end of the restricted feeding paradigm, and found that restricted feeding induced similar reduction of blood glucose in WT and KO mice (2×2 ANOVA: effect of genotype, *p* = 0.216, effect of feeding condition, *p* = 0.002, genotype x feeding condition, *p* = 0.234, Figure 1D).

### KO mice exhibit enhanced food-anticipatory activity

**Figure 2:**
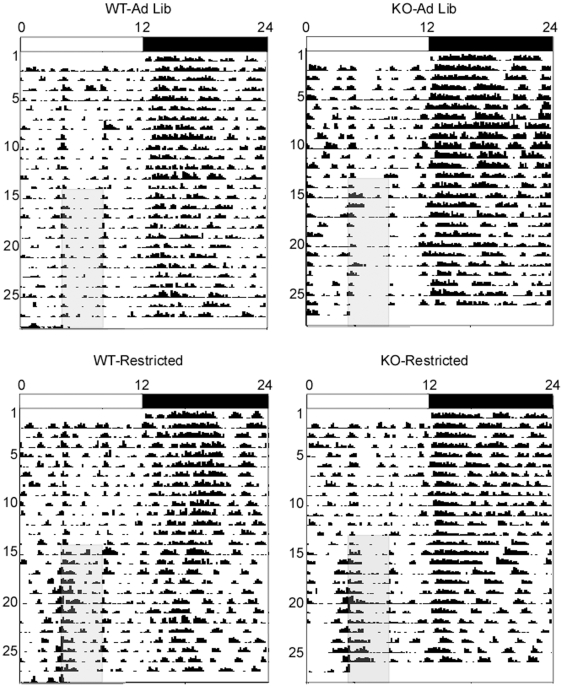
Representative actograms of WT and KO mice under *ad libitum* feeding conditions or a restricted feeding schedule. For all actograms in this study, each horizontal line represents a single 24-hour day, with the next day plotted on the next line. Vertical deflection reflect locomotor activity. The light/dark cycle is indicated by white/black bars above, the restricted feeding schedule and corresponding time window for *ad libitum*-fed control groups are indicated by gray shading.

**Figure 3:**
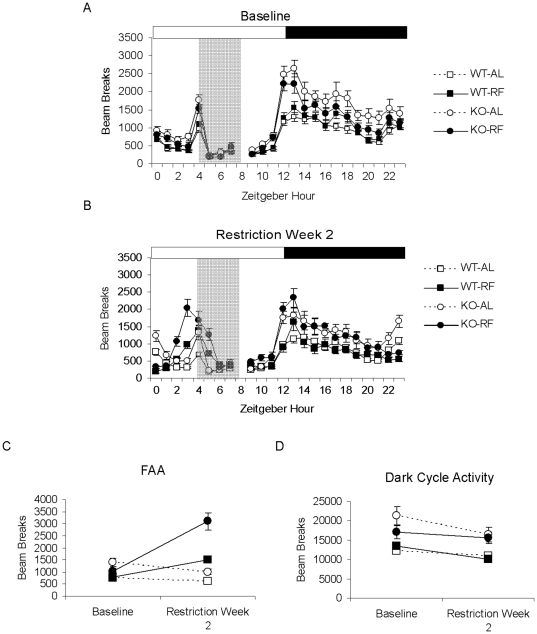
KO mice exhibit enhanced food-anticipatory activity in a standard LD schedule. A. 24-hour activity profile averaged over five days of baseline *ad libitum* feeding conditions for WT (squares) and KO (circles) mice assigned to either *ad libitum* (white) or restricted feeding (black) groups. B. 24-hour activity profile averaged over the second week of restricted feeding. C. Locomotor activity during the two hours preceding food availability, averaged over the baseline period and the second week of restricted feeding. D. Locomotor activity during the dark portion of the light/dark cycle, averaged over the baseline period and second week of restricted feeding. All values represent means ±S.E.M.

To determine whether FAA is differentially impacted in KO animals compared to WT mice, we examined FAA (locomotor activity that occurs in the two hours leading up to the daily feeding time) in response to a daytime restricted feeding schedule (Figure 2). Under baseline conditions, all experimental groups exhibited a spike in locomotor activity at ZT4, denoting behavioral activation in response to the daily food exchange (Figure 3A). However, baseline locomotor activity at ZT3 and at all other time points in the light cycle were minimal for all groups (Figure 3A). During the second week of restricted feeding, the restricted KO mice demonstrated robust enhancement of FAA compared to restricted WT mice (2×2×2 RM ANOVA: experiment phase (RW2 vs. Baseline), *p* = 0.002, experiment phase x genotype, *p* = 0.004, experiment phase x feeding condition, *p* = <0.001, experiment phase x genotype x feeding condition, *p* = 0.001, Figure 3B, 3C).

5-HT2CR null mutant mice are hyperactive at dark cycle onset and exhibit enhanced locomotor responses to contextual novelty [31]. To examine the possibility that the enhanced locomotor response of KO mice to restricted feeding is secondary to non-specific hyperactivity, we analyzed locomotor activity occurring during the dark portion of the light/dark cycle, which is a time of day associated with high locomotion unrelated to food entrainment. We did not observe enhanced locomotor activity at this time point in KO mice in response to restricted feeding (2×2×2 RM ANOVA: experiment phase (RW2 vs. Baseline), *p* = 0.001, experiment phase x genotype, *p* = 0.590, experiment phase x feeding condition, *p* = 0.683, experiment phase x genotype x feeding condition, *p* = 0.071, Figure 3D). Furthermore, analysis of all other time points (with adjusted *p* values using Bonferroni correction) confirmed that the locomotor enhancement in restricted KO mice occurs only during the FAA period, with no significant interaction between genotype and feeding condition at any other time points (Figure S1, Table S1).

### Enhanced food-anticipatory activity of KO mice persists during two days of total food deprivation

**Figure 4:**
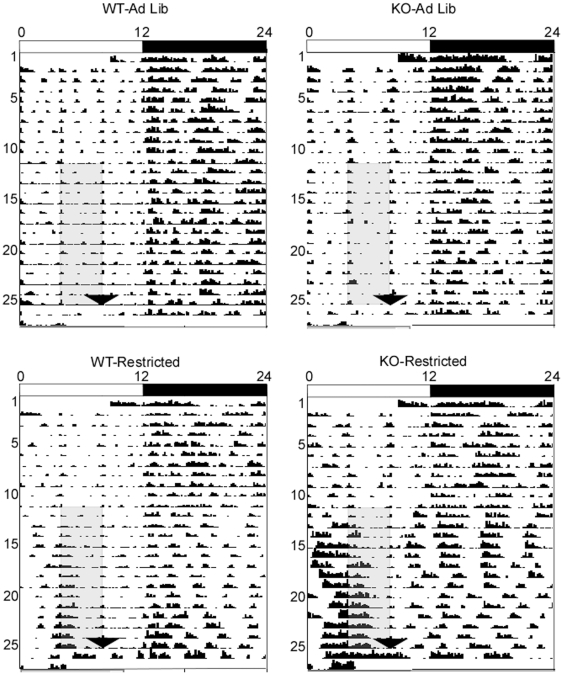
Representative actograms of WT and KO mice under *ad libitum* feeding conditions or a restricted feeding schedule with two additional days of food deprivation. The light/dark schedule is indicated by white/black bars above, the restricted feeding schedule and corresponding time window for *ad libitum*-fed control groups are indicated by gray shading. The start of total food deprivation is indicated by inverted triangles on the bottom right-hand corner of the shaded rectangles.

**Figure 5:**
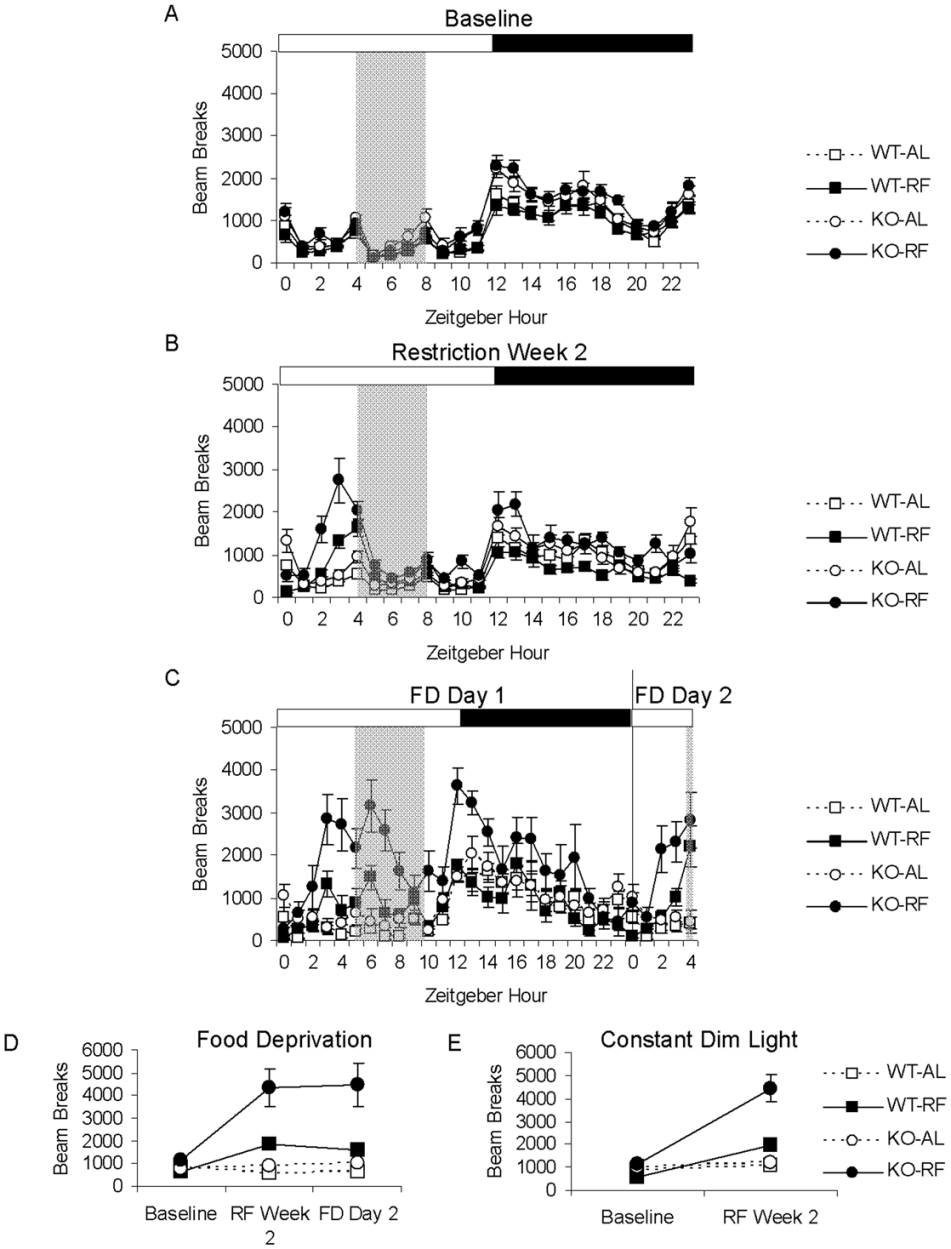
Enhanced FAA in KO mice persists after two days of food deprivation and in the absence of light cues. 24-hour activity profiles of WT (squares) or KO (circles) assigned to *ad libitum* (white symbols) or restricted feeding (black) groups, averaged over A. baseline B. second week of restricted feeding C. first and second day of total food deprivation. D. FAA averaged over the baseline period, second week of restricted feeding, and during the second day of food deprivation. E. FAA averaged over the baseline period and the second week of restricted feeding in constant dim red light. The light/dark cycle is indicated by white/black bars above, the restricted feeding schedule and corresponding time windows for *ad libitum* control groups and during the food deprivation period are indicated by gray shading. Values represent means ±S.E.M.

It is generally agreed that FAA arises as the behavioral output of a food-entrainable circadian oscillator (FEO). However, in the absence of an FEO, FAA could conceivably be stimulated each day by energy depletion and terminated by feeding, a so-called ‘hourglass’ process [3]. Hourglass timers must be reset each day and do not produce continued oscillations under constant conditions. To examine the possibility that FAA of KO mice reflects an hourglass mechanism, we subjected WT and KO mice to a restricted feeding paradigm with two additional days of total food deprivation following the 14 days of restricted feeding (Figure 4,5A–D). In these conditions, FAA developed in a similar manner as observed previously, with significant enhancement in restricted KO compared to WT mice during the second week of restricted feeding (2×2×2 RM ANOVA: experiment phase (RW2 vs. Baseline), *p*<0.001, experiment phase x genotype, *p* = 0.005, experiment phase x feeding condition, *p*<0.001, experiment phase x genotype x feeding condition, *p* = 0.038, Figure 5D). For the first day of food deprivation, we observed hyperactivity in restricted KO not only during the FAA time window but also during the expected mealtime and into the early portion of the dark cycle (Figure 5C), which is consistent with previous findings of general hyperactivity in response to fasting [30]. However, we found that the hyperactive response of restricted KO mice had normalized by the later portion of the dark cycle (Figure 5C), and that FAA persisted on the second day of food deprivation without corresponding hyperactivity at other time points (2×2×2 RM ANOVA: experiment phase (2nd day Food Deprivation vs. Baseline), *p*<0.001, experiment phase x genotype, *p* = 0.004, experiment phase x feeding condition, *p*<0.001, experiment phase x genotype x feeding condition, *p* = 0.056, Figure 5C, 5D). These results indicate that FAA of KO mice does not reflect an hourglass mechanism.

### Enhanced food-anticipatory activity of KO mice does not require light cues

**Figure 6:**
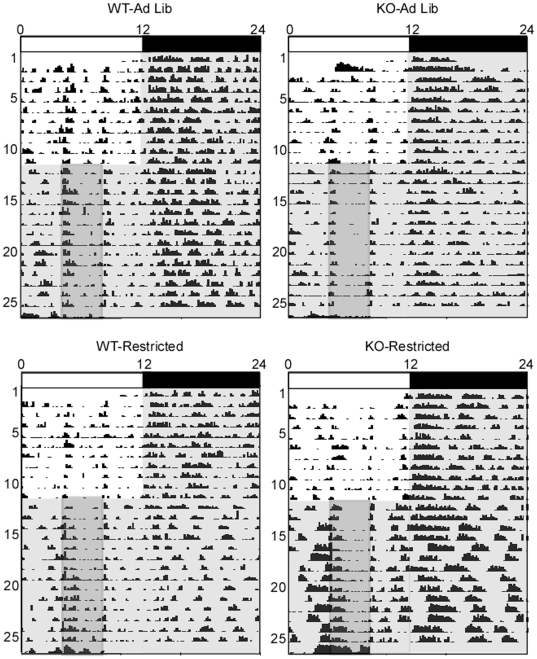
Representative actograms of WT and KO mice under *ad libitum* feeding conditions or a restricted feeding schedule conducted in constant dim red light. All animals were initially housed in a standard light/dark cycle (indicated by white/black bars above) with *ad libitum* food access, then switched to constant dim light (indicated by gray shading) at ZT12 the day prior to the start of the restricted feeding schedule. The restricted feeding schedule and corresponding time window for *ad libitum*-fed control are indicated by dark gray shading.

Many studies have shown that FAA occurs in the absence of photic cues such as light/dark schedules. Moreover, light of sufficient intensity (such as during the light portion of the light/dark cycle) suppresses locomotor activity in nocturnal rodents in a process known as light masking [33]. Therefore, it is possible that the enhanced FAA of KO mice may be due to decreased photic masking in KO animals, allowing enhanced expression of daytime FAA relative to WT mice. To determine whether the enhanced FAA phenotype demonstrated by KO mice is due to altered sensitivity to light, we compared levels of FAA in WT and KO mice in animals subjected to a restricted feeding schedule in constant dim red light (Figure 6). In these conditions, FAA developed in a similar manner as in a standard light/dark schedule, with significantly enhanced FAA in KO mice compared to WT by the second week of restricted feeding (2×2×2 RM ANOVA: experiment phase (RW2 vs. Baseline), *p*<0.001, experiment phase x genotype, *p* = 0.013, experiment phase x feeding condition, *p*<0.001, experiment phase x genotype x feeding condition, *p* = 0.009, Figure 5E).

### Restricted feeding induces similar c*-fos* gene expression in hypothalamic nuclei of WT and KO mice

**Figure 7:**
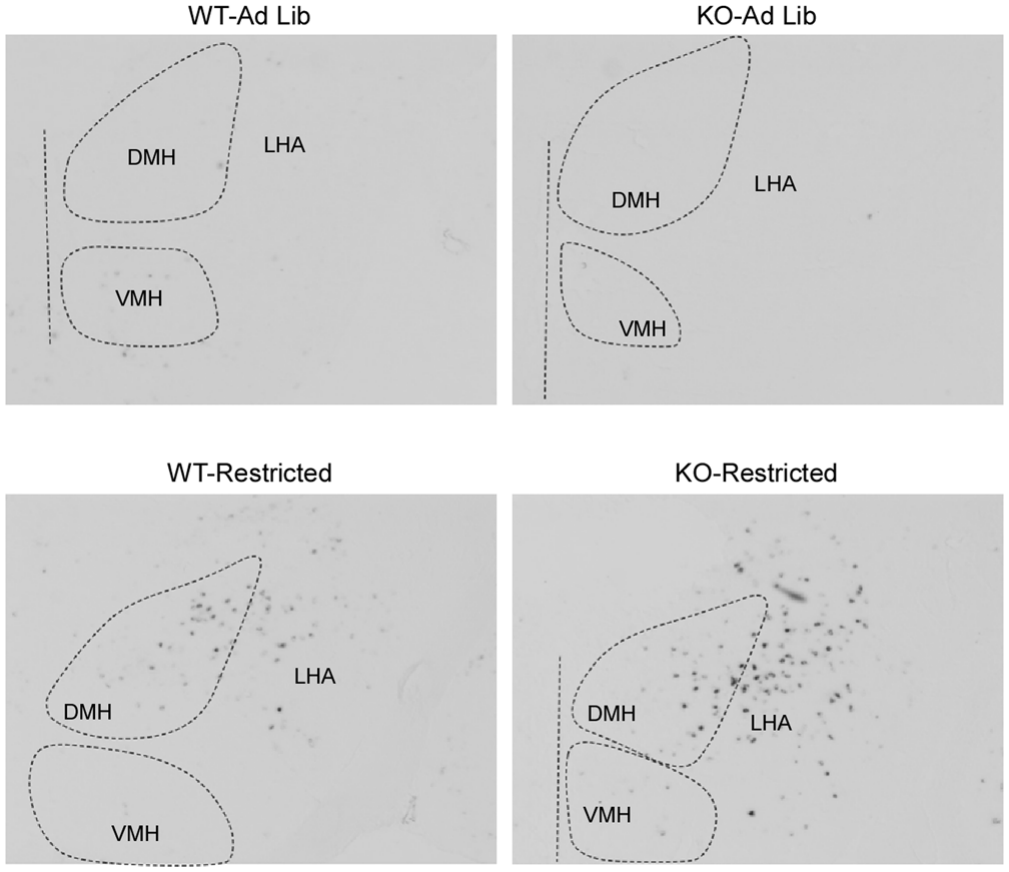
Representative photomicrographs of hypothalamic DMH/LHA c-*fos* expression at ZT4.

**Figure 8:**
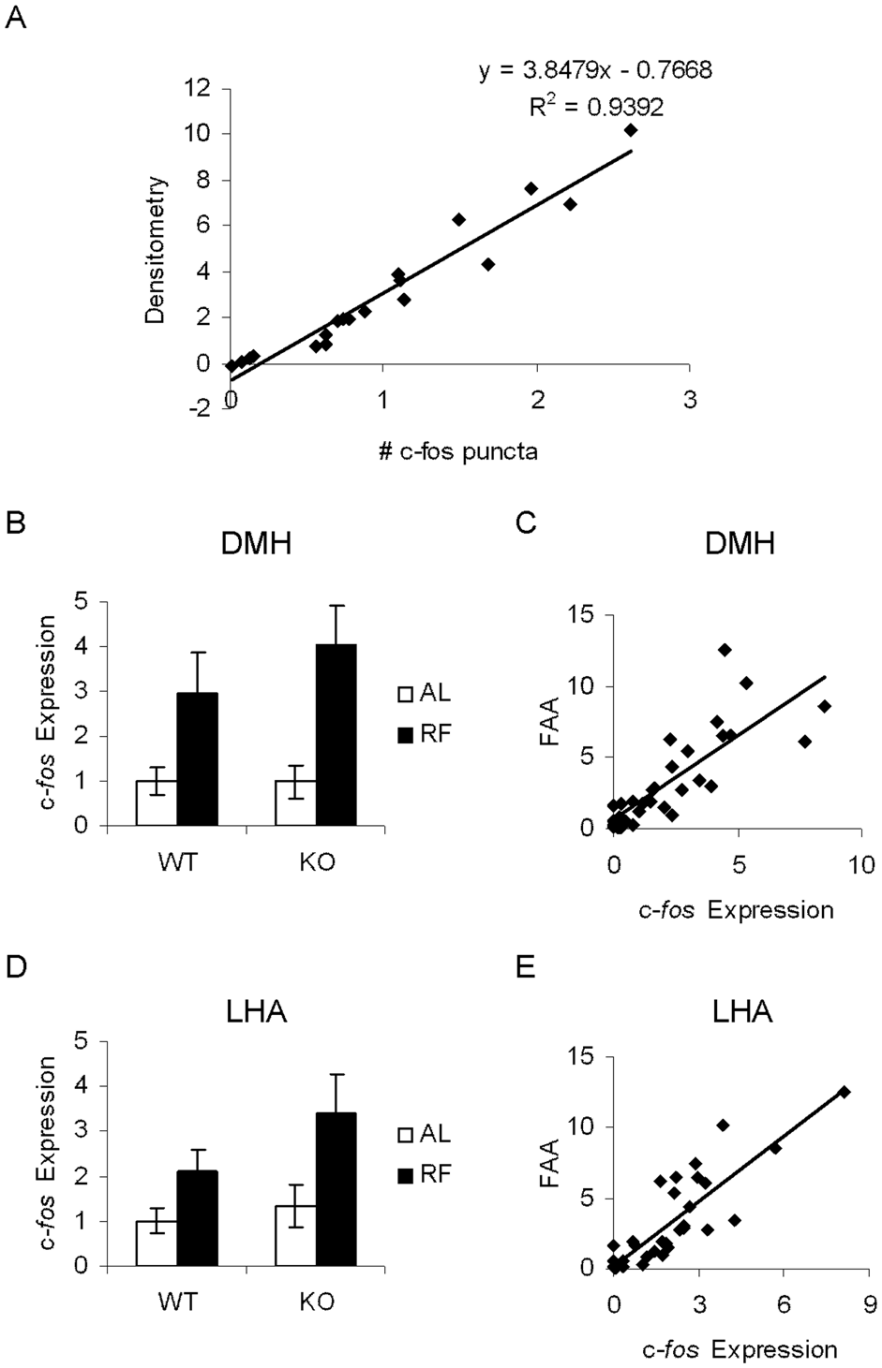
Restricted feeding induces c-*fos* expression in the DMH and LHA at ZT4. A. Correlation of ZT4 DMH c-*fos* densitometry values with number of c-*fos* puncta. B. Quantitation of ZT4 DMH c-*fos* mRNA. Bar graphs represent c-*fos* expression levels normalized to the WT-Ad Lib average value, expressed as means ±S.E.M. White bars indicate *ad libitum* feeding conditions, black bars indicate restricted feeding conditions. B. Correlation of locomotor activity to DMH c-*fos* gene expression. For correlation graphs, locomotor activity levels and c-*fos* expression levels are normalized to the WT-Ad Lib average values. C. Quantitation of ZT4 LHA *c*-*fos* mRNA. D. Correlation of locomotor activity to LHA c-*fos* gene expression.

To assess neural correlates of FAA in WT and KO mice, we analyzed c-*fos* gene expression in animals subjected to a restricted feeding schedule and sacrificed at ZT4 (*n* = 7−8 per group). To confirm our c-*fos* expression quantitation method by densitometry, we used linear regression analysis to correlate the densitometry values with manual counts of c-*fos* positive puncta in the DMH/LHA area, and found that c-*fos* densitometry values correlated very highly with manual c-*fos* puncta counts (R^2^ = .93, *p*<0.001, Figure 7, 8A). We found minimal c-*fos* gene expression in the DMH of *ad libitum*-fed animals but strong induction in restricted animals, with no significant phenotypic differences (2×2 ANOVA: effect of genotype, *p* = 0.436, effect of restriction, *p* = 0.001, genotype x feeding condition interaction, *p* = 0.42, Figure 7, 8B). Moreover, linear regression analysis revealed a significant correlation between ZT4 DMH *c*-*fos* expression and FAA (R^2^ = 0.65, p<0.001, Figure 8C.) Similar findings were observed for c-*fos* expression in the LHA. We found minimal c*-fos* gene expression in *ad libitum*-fed controls, and strong induction in restricted animals, without phenotypic differences (2×2 ANOVA: effect of genotype, *p* = 0.17, effect of feeding condition, *p* = 0.009, genotype x feeding condition interaction, *p* = 0.427, Figure 7, 8D). Moreover, there was a significant correlation between ZT4 LHA c-*fos* expression and FAA (R^2^ = 0.61, p<0.001, Figure 8E.)

**Figure 9:**
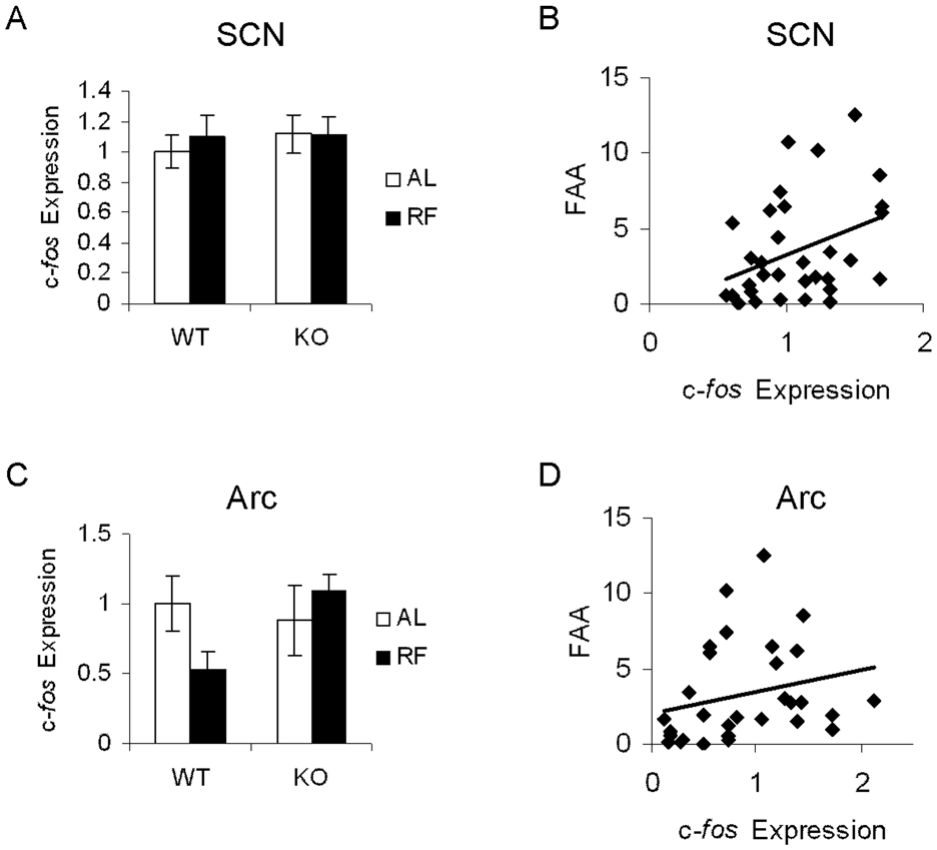
Effect of restricted feeding on SCN or ARC c-*fos* expression. A. Quantitation of ZT4 SCN c-*fos* mRNA. Bar graphs represent c-*fos* expression levels normalized to the WT-Ad Lib average value, expressed as means ±S.E.M. White bars indicate *ad libitum* feeding conditions, black bars indicate restricted feeding conditions. B. Correlation of locomotor activity to SCN c-*fos* gene expression. For correlation graphs, locomotor activity levels and c-*fos* expression levels are normalized to the WT-Ad Lib average values. C. Quantitation of ZT4 ARC *c*-*fos* mRNA. D. Correlation of locomotor activity to ARC c-*fos* gene expression.

The arcuate nucleus and SCN are proposed to provide afferent signals to the DMH, conveying information related to energy status information and circadian time [34]. However, we found no evidence that the arcuate nucleus or SCN are activated during FAA. We found modest c-*fos* gene expression in the SCN, with no effect of feeding condition or genotype (2×2 ANOVA: effect of genotype, *p* = 0.605, effect of feeding condition, *p* = 0.741, genotype x feeding condition interaction, *p* = 0.664, Figure S2, 9A). For the arcuate nucleus, there was a trend for reduced c-*fos* expression in restricted WT compared to *ad libitum*-fed WT controls (two-tailed *t*-test, *p* = 0.08), but no effect of restricted feeding in KO mice (Figure S3, 9C). Furthermore, there were no phenotypic differences between *ad libitum*-fed WT and KO animals (Figure 7C, 9C). Linear regression analysis revealed a modest correlation between SCN c-*fos* expression and FAA (R^2^ = 0.13, p = 0.044, Figure 9B), and no correlation between arcuate c-*fos* expression and FAA (R^2^ = 0.06, p = 0.203, Figure 9D).

### Phenotypic differences in c-*fos* induction by restricted feeding in extrahypothalamic regions

**Figure 10:**
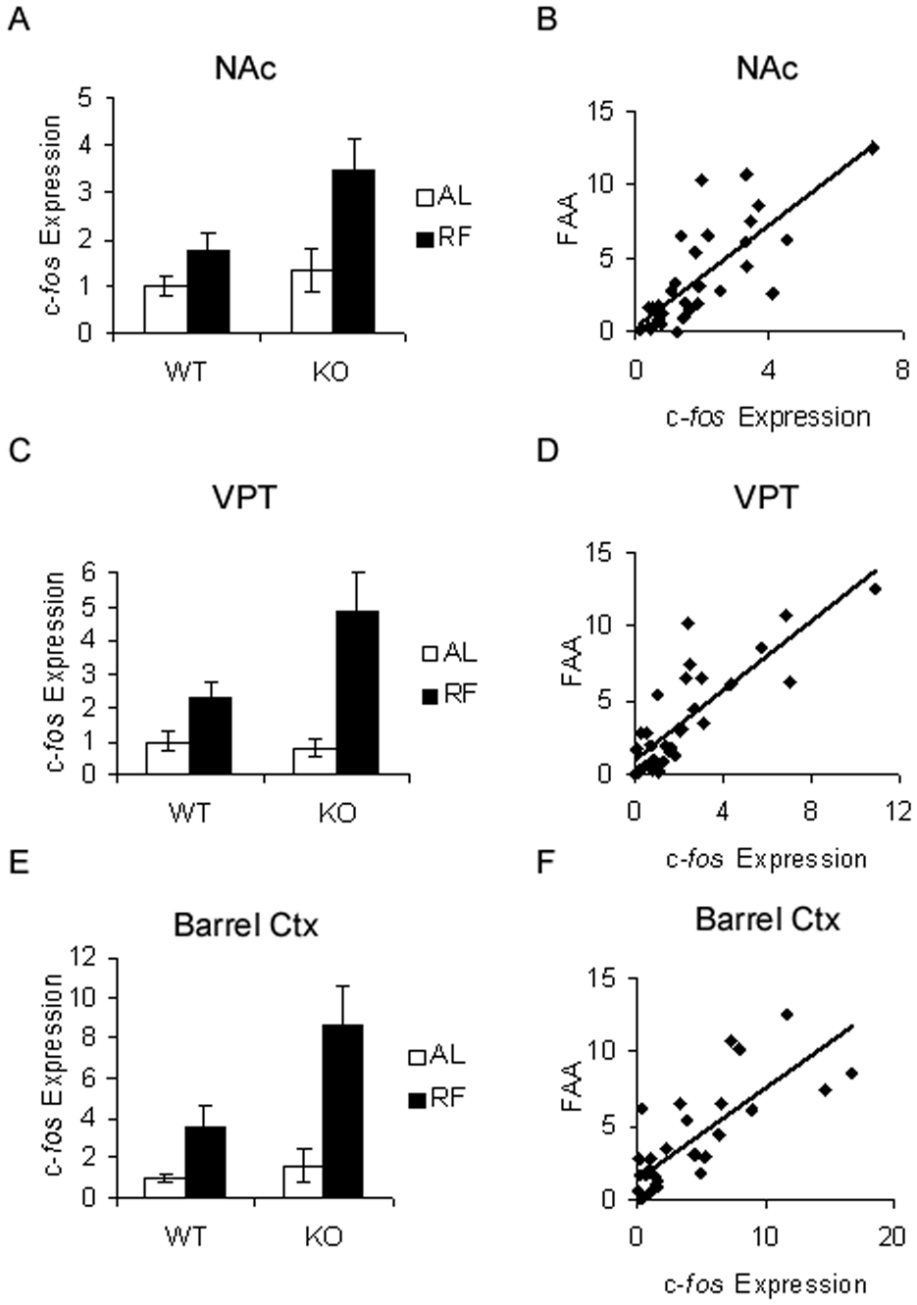
Enhanced c-*fos* induction by restricted feeding in extrahypothalamic regions of KO mice. A. Quantitation of ZT4 NAc c-*fos* mRNA. Bar graphs represent c-*fos* expression levels normalized to the WT-Ad Lib average value, expressed as means ±S.E.M. White bars indicate *ad libitum* feeding conditions, black bars indicate restricted feeding conditions. B. Correlation of locomotor activity to NAc c-*fos* gene expression. For correlation graphs, locomotor activity levels and c-*fos* expression levels are normalized to the WT-Ad Lib average value. C. Quantitation of ZT4 VPT *c*-*fos* mRNA. D. Correlation of locomotor activity to VPT c-*fos* gene expression. E. Quantitation of ZT4 barrel cortex *c*-*fos* mRNA. F. Correlation of locomotor activity to barrel cortex c-*fos* gene expression.

**Figure 11:**
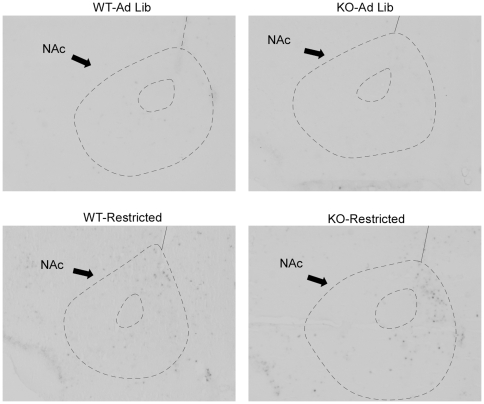
Representative photomicrographs of c-*fos* expression in the NAc at ZT4.

**Figure 12:**
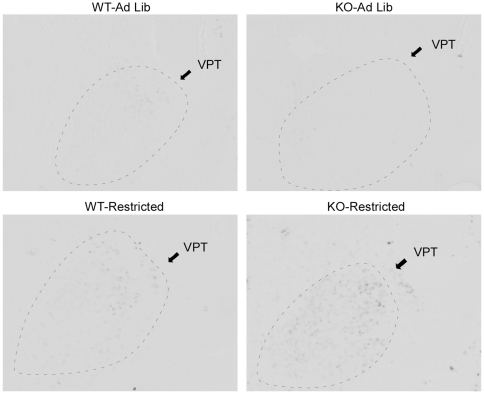
Representative photomicrographs of c-*fos* expression in the VPT at ZT4.

**Figure 13:**
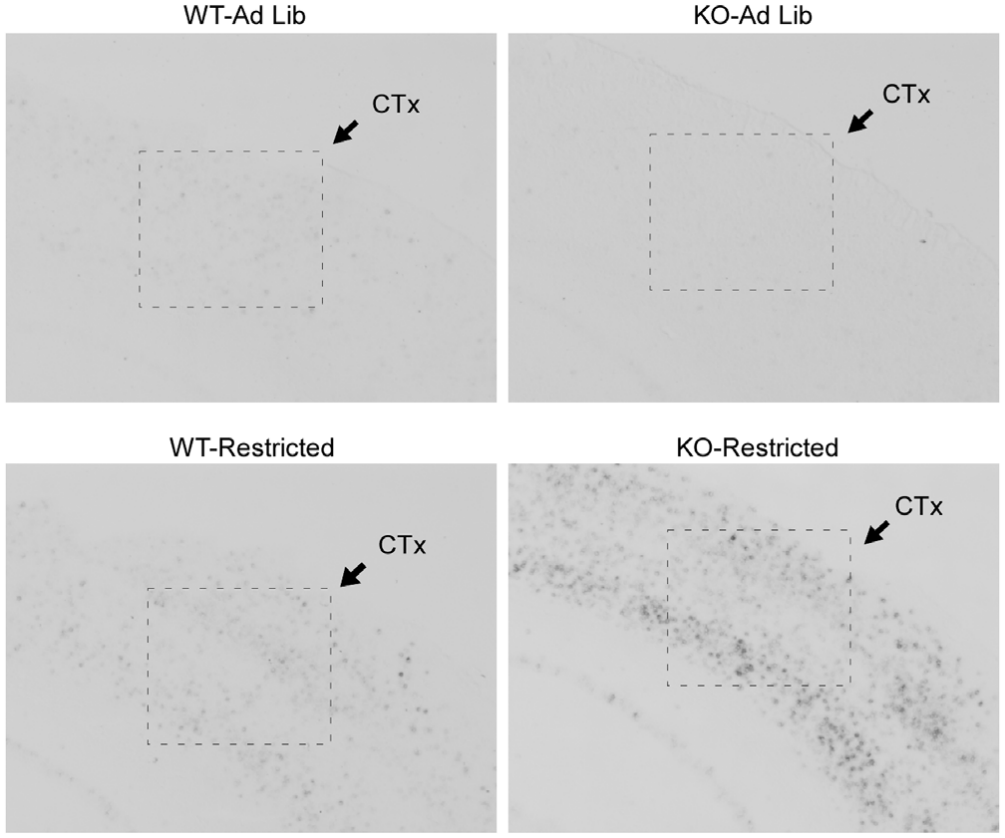
Representative photomicrographs of c-*fos* expression in the barrel cortex at ZT4.

In addition to the hypothalamic regions described above, the NAc has been implicated in the regulation of FAA. In the NAc, restricted feeding induced c-*fos* expression modestly in WT mice, with enhanced induction in KO mice relative to WT (Mann Whitney U: effect of genotype *p* = 0.09, effect of feeding condition *p* = 0.002, restricted KO>restricted WT, *p* = 0.012, Figure 10A, 11). Moreover, there was a significant correlation between NAc c-*fos* expression and FAA (R^2^ = 0.60, p<0.001, Figure 10B). In addition to the NAc, a survey of additional extrahypothalamic sites was conducted for regions that demonstrated c-*fos* induction by restricted feeding with enhanced induction in restricted KO relative to restricted WT. Several cortical regions, but not the dorsal striatum, demonstrated strong c-*fos* induction by restricted feeding without phenotypic differences (Table S2). Furthermore, two additional regions exhibited c-*fos* induction by restricted feeding with enhanced induction in restricted KO mice relative to WT: the ventral posterior area of the thalamus (VPT), and the sensory barrel cortex. In the VPT, restricted feeding induced c-*fos* expression modestly in WT mice, with enhanced induction in KO mice (2×2 ANOVA: effect of genotype *p* = 0.06, effect of feeding condition *p*<0.001, genotype x feeding condition interaction *p* = 0.026, Figure 10C, 12). Moreover, there was a significant correlation between VPT c-*fos* expression and FAA (R^2^ = 0.67, p<0.001, Figure 10D). In the sensory barrel cortex, restricted feeding strongly induced c-*fos* expression in both genotypes, with a trend towards enhanced induction in restricted KO mice (Mann-Whitney U: effect of genotype *p* = 0.439, effect of feeding condition *p* = 0.001, KO-Restricted>WT-Restricted, *p* = 0.059, Figure 10E, 13). Moreover, there was a significant correlation between barrel cortex c-*fos* expression and FAA (R^2^ = 0.61, p<0.001, Figure 10F).

### Neural correlates of FAA do not correlate with non-specific locomotor activity

To determine whether neural activation in the above regions correlate specifically with FAA or reflect general locomotor activity, we assessed ZT13 c-*fos* expression in the DMH, LHA, NAc, and barrel cortex. ZT13 c-*fos* expression reflects the first hour of the dark cycle—which is a time point at which mice exhibit a significant increase in locomotor activity unrelated to food anticipation, and during which KO mice are significantly hyperactive compared to WT. We found no significant correlations between locomotor activity occurring in the first hour of the dark cycle and c-*fos* gene expression in any of the regions tested (DMH correlation: R^2^ = 0.051, *p* = 0.338, Figure S4A; LHA correlation: R^2^ = 0.109, *p* = 0.156, Figure S4B; barrel cortex correlation: R^2^ = 0.085, *p* = 0.211, Figure S4C; NAc correlation: R^2^ = 0.019, *p* = 0.567, Figure S4D).

## Discussion

We find that, in response to a temporally restricted feeding schedule, 5-HT2CR null mutant mice exhibit enhanced FAA relative to wild-type mice. In addition, the enhanced FAA phenotype of KO mice persists during two days of total food deprivation and also in constant dim red light conditions, indicating that FAA in these mice is not driven by an hourglass mechanism or attributable to altered sensitivity to light. Furthermore, enhanced FAA of KO mice is associated with enhanced c-*fos* induction in the NAc and other extrahypothalamic regions but not with phenotypic enhancement of hypothalamic c-*fos* induction. These findings support the notion that enhanced FAA in KO mice reflects a behavioral manifestation of a food-driven circadian oscillator and suggest that 5-HT2CRs gate food-anticipatory activity through mechanisms involving extrahypothalamic neural pathways.

It should be noted that 5-HT2CR null mutant mice exhibit enhanced locomotor responses to a number of stimuli, such as dark cycle onset, novelty, and cocaine administration [31], [35]. This raises the possibility that the enhanced FAA of KO mice may reflect a non-specific locomotor response to restricted feeding. However, we find that the enhanced locomotor response of KO mice to restricted feeding occurs only during the FAA time window and not at any other time of day, indicating that the differential response of KO mice to restricted feeding cannot be attributed solely to non-specific locomotor enhancement. Furthermore, our results indicated that in KO mice, neural correlates of enhanced FAA are distinct from neural correlates of general hyperactivity. In our study, several brain regions exhibited significant increases in c-*fos* expression at ZT4 with restricted feeding and enhanced activation in restricted KO compared to restricted WT; moreover, ZT4 c*-fos* expression levels in these regions were highly correlated with FAA. By contrast, ZT13 c-*fos* expression levels in the same regions did not correlate with locomotor activity in the early portion of the dark cycle, another time of robust hyperactivity in KO mice relative to WT. These findings further support the notion that mechanisms underlying enhanced FAA in KO are distinct from general hyperactivity. It is possible that in KO mice, enhanced FAA may be attributed either to enhancement of the FEO or to enhancement of FEO-efferent locomotor response pathways.

Hypothalamic signaling has been strongly implicated in the regulation of FAA. The DMH has been proposed as a component of the FEO, based on lesion studies [19] and the recent finding that viral-mediated restoration of the clock gene BMAL1 selectively into the DMH of BMAL1 KO animals restored food-entrainable circadian rhythms but not light-entrainable rhythms [36]. However, other studies do not support a central role for the DMH or BMAL1 in the regulation of FAA [22], [23], [24], [37], [38], [39]. The LHA has also been proposed to modulate the expression of FAA, based on findings that this region exhibits c-*fos* induction coincident with the timing of FAA [12] and on the results of lesion experiments that report attenuated FAA in mice with genetic ablations of LHA orexin neurons [14], [17]. However, other studies report normal FAA after LHA lesions in at least one measure of food anticipatory behavior [40]. Our results demonstrate no significant phenotypic differences in DMH and LHA c-*fos* expression despite marked phenotypic differences in the magnitude of FAA, indicating that differential activation of these regions is not associated with enhanced FAA in KO animals. However, these findings do not entirely exclude the possibility that phenotypic differences in DMH or LHA function contribute to the enhanced FAA exhibited by KO mice.

Hypothalamic leptin signaling pathways have also been implicated in the regulation of FAA, as shown by the modulatory effects of arcuate NPY lesions [41] and genetic ablation of melanocortin-3 receptors [42]. Our results show a modest trend towards reduced arcuate c-*fos* expression with restricted feeding in WT but not KO mice. It is possible that phenotypic differences in arcuate function may contribute to enhanced FAA in KO mice.

Studies investigating the neural substrates underlying FAA have largely focused on hypothalamic nuclei, and the contributions of extrahypothalamic regions to this phenomenon have been less thoroughly examined. The NAc has been proposed as a crucial region in which homeostatic signaling, motivational state, and behavioral expression are integrated [43], [44]. In rats, combined ablation of both the core and shell regions of the NAc does not prevent the generation of FAA or the persistence of FAA rhythms during total food deprivation [21]. However, targeted lesions of the NAc core versus shell produce opposite effects on FAA [18]. Furthermore, feeding paradigms that induce FAA have been shown to induce c-*fos* and to shift *Per1* rhythms in the NAc [13], suggesting a possible role for the NAc in the regulation of FAA. In this study, c-*fos* expression in the NAc is induced by restricted feeding, enhanced in restricted KO animals relative to restricted WT, and significantly correlated with FAA. By contrast, NAc c-*fos* expression levels do not correlate with locomotor activity occurring at dark cycle onset. These results indicate that phenotypic differences in NAc c-*fos* expression may contribute to the enhanced FAA of KO mice.

Our observation of enhanced activation of the NAc in restricted KO mice relative to restricted WT is notable, given the known inhibitory role of 5-HT2CRs on NAc function. Pharmacological and genetic studies reveal that NAc 5-HT2CRs exert a tonic inhibitory influence on NAc dopamine neurotransmission [45], [46], [47]. In addition, 5-HT2CR null mutant mice exhibit enhanced behavioral responses to novelty and cocaine administration, effects that have been attributed to enhanced dopamine neurotransmission in the NAc [31]. Our study raises the possibility that 5-HT2CRs modulate FAA by gating the sensitivity of the NAc to signals related to food entrainment.

Our observation of enhanced NAc activation correlated with enhanced FAA raises the possibility that FAA may be regulated by mesolimbic dopamine systems. To our knowledge, the role of the ventral tegmental area (VTA, source of dopaminergic input to the NAc and other limbic regions) in the regulation of FAA has not been characterized. Future analyses of c-*fos* expression in the VTA in response to temporally restricted feeding schedules may help dissect the contribution of 5-HT2C receptors and mesolimbic circuits to the regulation of food entrainable circadian rhythms.

Unlike the NAc, the VPT and barrel cortex have not been strongly implicated in the regulation of FAA. Neither region has been reported to receive afferents from the DMH or LHA, or to express c-*fos* in response to restricted feeding, therefore, the mechanisms by which VPT and barrel cortex are influenced by food entrainment are unclear. The VPT and barrel cortex represent the thalamic and cortical nodes of the sensory barrel system, which process whisker-mediated sensation in rats and mice [48]. It is possible that FAA causes secondary activation of these regions due to the increased sensory input associated with high levels of locomotor activity. However, our results demonstrate that barrel cortex c-*fos* expression levels were highly correlated with FAA but not with locomotor activity occurring at dark cycle onset. This indicates that activation of the sensory barrel system during FAA is not solely an indirect consequence of locomotor activity. The relationship of the sensory barrel system to FAA warrants future investigation.

In summary, our findings suggest a novel role for 5-HT2CRs in the regulation of food-anticipatory activity. Furthermore, c-*fos* gene expression studies in food-entrained WT and KO mice suggest a role for extrahypothalamic pathways in the regulation of FAA, and implicate 5-HT2CRs in the modulation of these processes. These results may help shed light on the neural mechanisms underlying the regulation of food anticipatory activity.

## Supporting Information

Figure S1 Demonstration that phenotypic effects of restricted feeding on locomotor activity is specific to FAA and not any other time points in KO mice.
(0.08 MB TIF)

Figure S2 Representative photomicrographs of c-fos expression in the SCN at ZT4.
(1.57 MB TIF)

Figure S3 Representative photomicrographs of c-fos expression in the arcuate nucleus at ZT4.
(1.57 MB TIF)

Figure S4 Neural correlates of FAA are not correlated with non-anticipatory locomotor activity. A–D. Correlations between ZT12–13 locomotor activity and ZT13 c-fos expression for A. DMH B. LHA C. Barrel Cortex D. NAc. For correlation graphs, locomotor activity levels and c-fos expression levels are normalized to the WT-Ad Lib average value.
(0.19 MB TIF)

Table S1 Summary of statistics for locomotor activity for all time bins from animals subjected to a restricted feeding schedule in a standard light/dark cycle.
(0.03 MB DOC)

Table S2 Summary of statistics for ZT4 c-fos gene expression in other extrahypothalamic brain regions.
(0.02 MB RTF)
