# Supplementary figures and images for: Enhanced Food Anticipatory Activity Associated with Enhanced Activation of Extrahypothalamic Neural Pathways in Serotonin2C Receptor Null Mutant Mice
Source: PLoS One. 2010 Jul 27;5(7):e11802. doi: 10.1371/journal.pone.0011802 (PMC2910710; doi:10.1371/journal.pone.0011802)

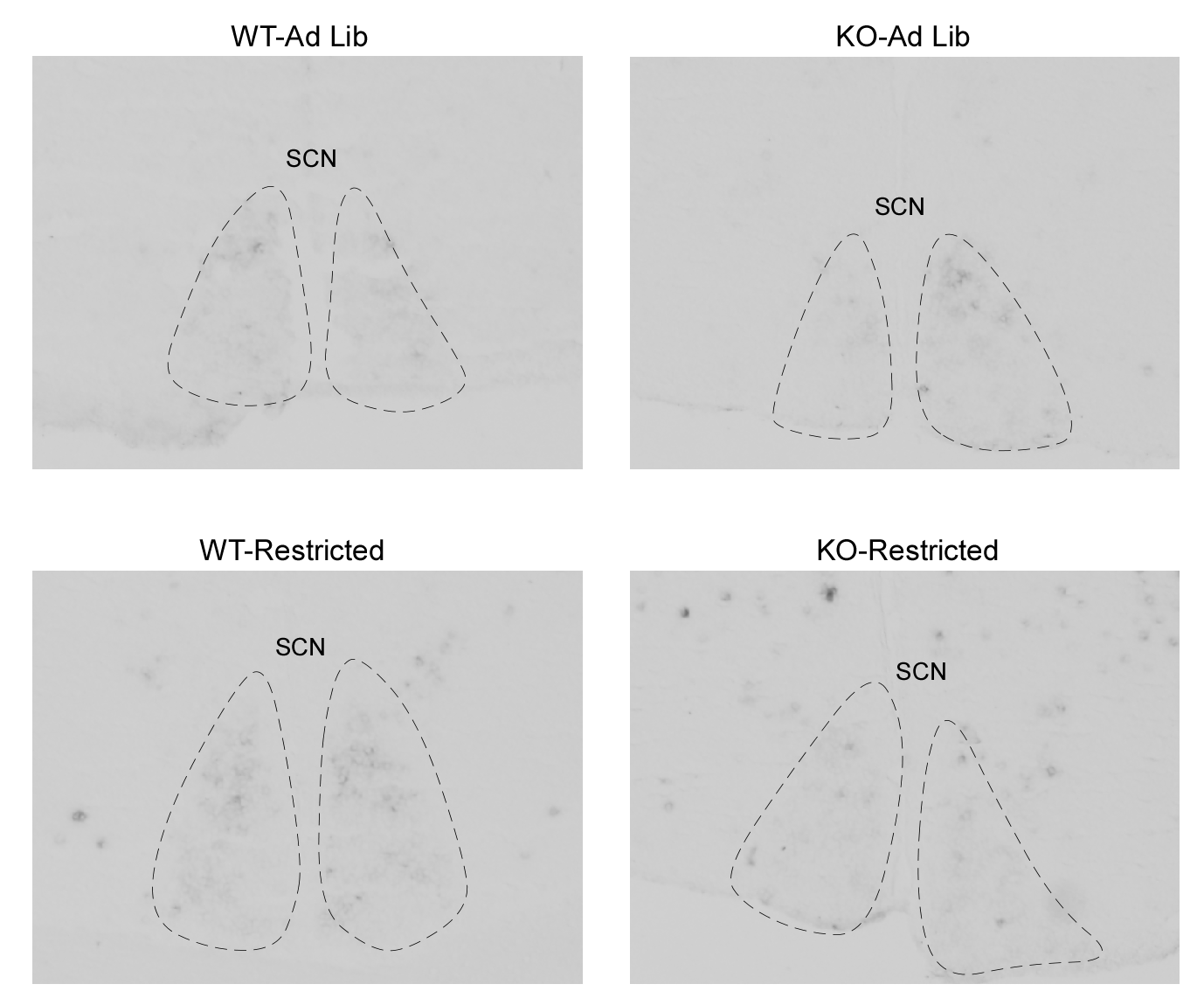

Supplement: Figure S2 — Representative photomicrographs of c-fos expression in the SCN at ZT4. (1.57 MB TIF) [file pone.0011802.s002.tif]

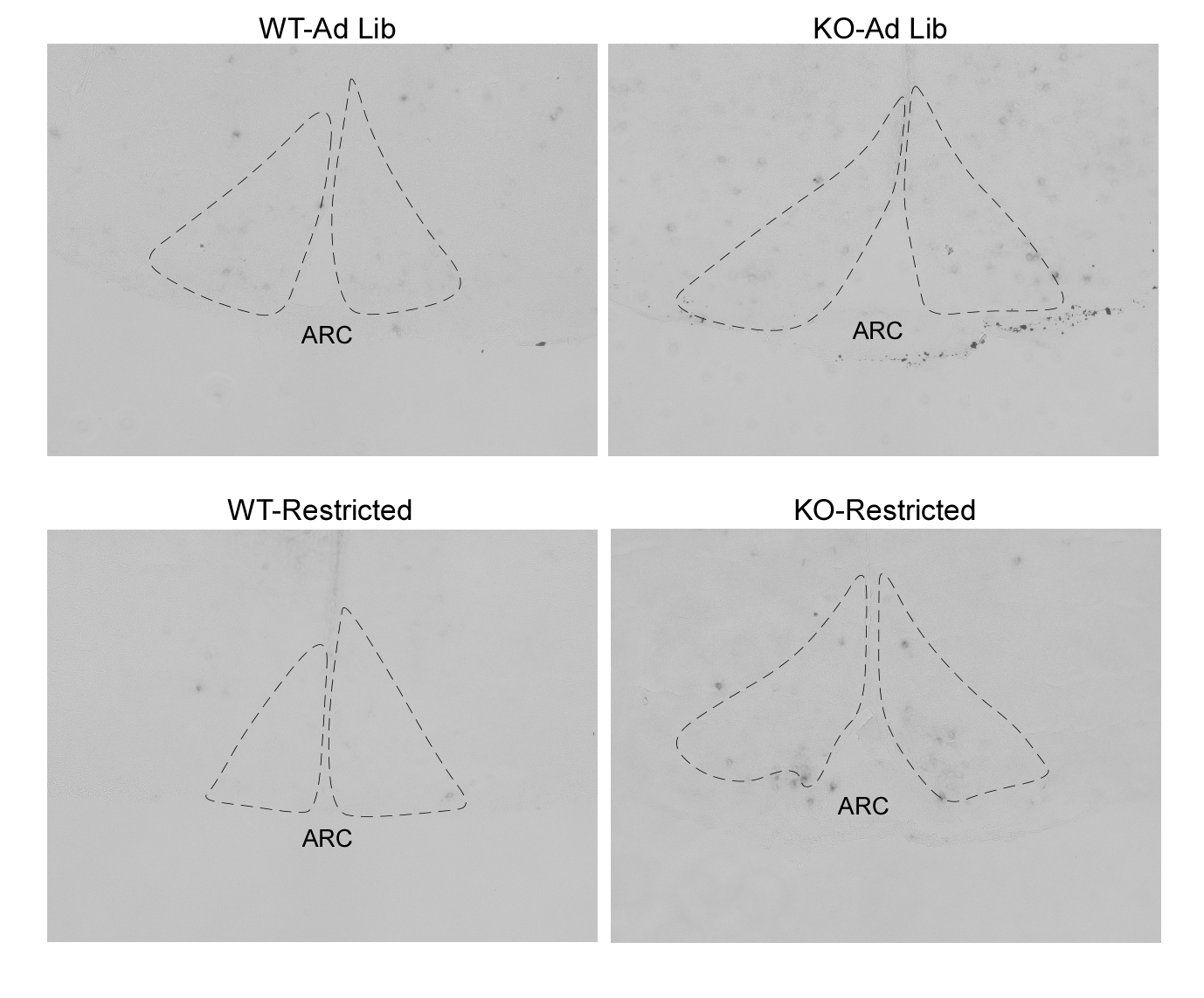

Supplement: Figure S3 — Representative photomicrographs of c-fos expression in the arcuate nucleus at ZT4. (1.57 MB TIF) [file pone.0011802.s003.tif]
