# Supplementary material for: Enhanced Food Anticipatory Activity Associated with Enhanced Activation of Extrahypothalamic Neural Pathways in Serotonin2C Receptor Null Mutant Mice
Source: PLoS One. 2010 Jul 27;5(7):e11802. doi: 10.1371/journal.pone.0011802 (PMC2910710; doi:10.1371/journal.pone.0011802)
Supplement: Table S1 — Summary of statistics for locomotor activity for all time bins from animals subjected to a restricted feeding schedule in a standard light/dark cycle. (0.03 MB DOC) [file pone.0011802.s005.doc]

| **TimeBin** | **Effect of genotype** | **Effect of feeding condition** | **genotype x feeding condition** |
| --- | --- | --- | --- |
| ZT0-2 | >0.1 | <0.001 | >0.1 |
| ZT2-4(FAA) | >0.1 | <0.001 | 0.003 |
| ZT4-6 | >0.1 | <0.001 | >0.1 |
| ZT6-8 | >0.1 | >0.1 | >0.1 |
| ZT8-10 | >0.1 | 0.012 | >0.1 |
| ZT10-12 | >0.1 | >0.1 | >0.1 |
| ZT12-14 | >0.1 | >0.1 | >0.1 |
| ZT14-16 | >0.1 | >0.1 | >0.1 |
| ZT16-18 | >0.1 | >0.1 | >0.1 |
| ZT18-20 | >0.1 | >0.1 | >0.1 |
| ZT20-22 | >0.1 | >0.1 | >0.1 |
| ZT22-24 | >0.1 | 0.012 | >0.1 |
