# Supplementary material for: Enhanced Food Anticipatory Activity Associated with Enhanced Activation of Extrahypothalamic Neural Pathways in Serotonin2C Receptor Null Mutant Mice
Source: PLoS One. 2010 Jul 27;5(7):e11802. doi: 10.1371/journal.pone.0011802 (PMC2910710; doi:10.1371/journal.pone.0011802)
Supplement: Table S2 — Summary of statistics for ZT4 c-fos gene expression in other extrahypothalamic brain regions. (0.02 MB RTF) [file pone.0011802.s006.rtf]

	Ad Lib	Restricted	                              p value	
	WT	KO	WT	KO	Geno	Feeding condition	Geno x feeding condition	
Retrosplenial Ctx	1.07±0.51	0.37±0.26	4.07±0.69	4.94±0.92	0.671	<0.001	0.561	
Parietal Ctx	0.80±0.40	0.58±0.21	6.16±2.62	6.34±0.86	0.749	0.004	0.868	
Piriform Ctx	2.65±1.20	2.78±1.08	6.59±1.87	7.08±1.70	0.839	0.018	0.906	
Striatum	0.02±0.02	0.26±0.26	0.60±0.23	0.51±0.32	0.762	0.104	0.500	
